# A spatio-temporal analysis of the magnitude and trend of land use/land cover changes in Gilgel Gibe Catchment, Southwest Ethiopia

**DOI:** 10.1016/j.heliyon.2024.e24416

**Published:** 2024-01-20

**Authors:** Zewde Alemayehu Tilahun, Yechale Kebede Bizuneh, Abren Gelaw Mekonnen

**Affiliations:** a Env't & Natural Resource Management, Dep't of Geography & Env'tal Studies, Arba-Minch University, Ethiopia; b Environmental Science, Dep't of Geography & Environmental Studies, Arba-Minch University, Ethiopia; c Environment & Natural Resources Management, Dep't of Geography & Environmental Studies, Arba-Minch University, Ethiopia

**Keywords:** Land use/land cover change, Forest depletion, Cultivated land expansion, Gilgel gibe

## Abstract

Analyzing alterations in land use/land cover is crucial for water Scientists, planners, and decision-makers in watershed management. This examination enables the development of effective solutions to mitigate the adverse impacts resulting from such changes. The focus of this research was analyzing alterations in land use/land cover within the Gilgel Gibe Catchment in 1991 – 2021. LULC data of 1991-2021 were derived from multispectral Landsat images. Data were also gathered using field observations and key informant interview. Data of LULC classes (1991–2021) were generated utilizing supervised classification with maximum likelihood algorithm of ENVI 5.1 and ArcGIS 10.5. Change detection analysis and accuracy assessment were done where accuracy levels all the study periods were > 85 %, and the overall Kappa statistics of the periods were > 0.89. Built-up area and cultivated land of the catchment are increasing with increasing magnitude of change; whereas, while forest cover and grazing land of the catchment are shrinking with declining magnitudes of change, shrubland covers and water body are declining with increasing magnitude of change in the catchment. The net increase in degraded land is a reflection of the increasing degradation of natural resources in the catchment. Swift escalation of population and the subsequent raising demand for farmland and forest and shrub (e.g. fuel-wood and construction) products, decline yield, unemployment and lack of alternative income source, and open access and limited conservation of resources are the principal factors for the dramatic shrinkages of grazing, forest, water body and shrubland resources. Thus, concerned bodies should take rehabilitation measures to restore degraded lands, improve production and yield of farmland by increasing improved farm-inputs and irrigation, and create employment and alternative income sources for the youth, women and the poor so as to ensure sustainable rural livelihoods and to curb the impacts on forest, shrubland and other resources.

## 1 Introduction

Processes influenced by human activities that are linked to alterations in land use/land cover (LU/LC) are rapidly and significantly reshaping the Earth's surface [[1], [2], [3]]. These changes have far-reaching consequences for the environment, including the loss of biodiversity, shifts in climate patterns, degradation of soil and water quality, and potential threats to food security [[4], [5], [6], [7], [8], [9], [10]]. The patterns, causes, and consequences of alterations in land use/land cover (LU/LC) exhibit variations depending on the specific regions and ecosystems, necessitating a thorough examination within their unique contextual frameworks. Notably, a significant global driver of LULC change involves the conversion of natural ecosystems such as grasslands, woodlands, and forests into agricultural land [1,6,7,[11], [12], [13], [14], [15], [16], [17], [18]]. with a particular emphasis on this trend in Africa (Brandt et al., 2018). The transformation of natural ecosystems into agricultural land negatively impacts essential ecosystem services like carbon capture, water control, and soil preservation. Each year, 16 million hectares of forest land are cleared globally, trigger an escalation in greenhouse gas emissions and a diminish in carbon storage, as noted by Ref. [14], and [19]. Over the past few years, there has been a rise in the transformation of African grasslands, woodlands, and other natural habitats into agricultural areas, impacting the water cycle and intensifying soil erosion. To establish land management practices and policies that are sustainable; capable of harmonizing the demands of food production and environmental preservation, it is imperative to comprehend the patterns, causes, and consequences of land use and land cover changes in Africa [20,21].

The predominant factor driving noteworthy alterations in land use and land cover (LULC) in Ethiopia over the last five decades has been the enlargement of farming land, as highlighted in studies by Refs. [10,[22], [23], [24], [25]]. These alterations have exacerbated the degradation of ecosystem services, encompassing soil preservation, carbon capture, biodiversity, and water control. Additionally, they have heightened the region's susceptibility to food poverty and climate change [26,27]. Several studies have indicated a potential enhancement in land quality and productivity, as observed in positive trends of land use/land cover changes in different regions of Ethiopia. Examples of these changes include the expansion of plantations and the reduction of bare land [28,29]. To establish sustainable land management techniques and policies that strike a harmonious balance between environmental protection and production of food, it is crucial to comprehend the scheme, causes, and consequences of land use/cover conversion (LU/LC) alterations in Ethiopia. Merely possessing an extensive understanding of land use and land cover (LULC) changes and the associated local factors, as indicated by Refs. [30,31], is insufficient for the development of plans and the evaluation of policies related to land. Furthermore, it is imperative to evaluate the repercussions of these changes on land health, ecosystems, as well as human livelihoods and well-being, as emphasized by Refs. [32,33]. To deal with the escalating requirements for fundamental human needs and welfare, the selection, planning, and execution of diverse development projects necessitate crucial insights into land use, land cover, and opportunities for their optimal utilization [34]. According to the research by Ref. [35], the results suggest that although remote sensing mapping of land use/cover change (LU/LC) delivers quantitative descriptions of alterations, it falls short in clarifying or illuminating the connections between the variables driving the changes and their underlying causes. Field surveys and interviews serve as vital supplementary methods to uncover the fundamental causes and consequences of Land Use/Land Cover (LU/LC) changes. They are also essential for collecting the viewpoints and local expertise of land users and managers.

The environment and civilization are significantly affected by LULC dynamics, which are the outcome of intricate interconnections between natural and human systems. To formulate appropriate strategies and plans for managing land, it is crucial to comprehend the rates, extents, patterns, causes, and implications of Land Use/Land Cover (LU/LC) sways at the catchment level. In emerging nations characterized by significant population pressure and resource degradation, there is a limited cognizance of the dynamics and underlying causes of changes at the watershed level [36] assert that the Gilgel Gibe catchment in the southwest of Ethiopia holds significant importance, is intensively utilized, and stands as an environmentally fragile catchment. Situated within the Omo Gibe basin, it contributes essential ecological services, irrigation water, and hydroelectricity to millions of people. Primarily propelled by the growth of farming, deforestation, urbanization, and infrastructural development, the catchment has undergone rapid and extensive Land Use and Land Cover (LULC) changes in the past few decades. The sustained functionality of the Gilgel Gibe Reservoir and the well-being of the surrounding populations face threats from soil erosion, nitrogen depletion, biodiversity loss, and deteriorating water quality resulting from these changes.

While addressing this issue is crucial, there is an incomplete understanding of the Land Use and Land Cover (LULC) dynamics and underlying causes in the Gilgel Gibe catchment. The precision and significance of the results are constrained by the fact that previous studies focused on specific sub-basins or utilized low-resolution data. No research has amalgamated social survey methods with remote sensing to evaluate both natural and human elements contributing to Land Use and Land Cover (LULC) changes. Therefore, to formulate appropriate land management plans and regulations, it is crucial to comprehend the intricate and dynamic relationship between the primary and indirect causes of Land Use and Land Cover (LULC) fluctuations. Hence, a comprehensive and integrated approach is necessary for evaluating Land Use and Land Cover (LULC) movements and their impacts in the Gilgel Gibe catchment. In the present study, the examination focuses on the changes in Land Use and Land Cover (LULC) and their impacts on the Gilgel Gibe basin, acknowledged as one of Ethiopia's most crucial and vulnerable basins. The study adopts a mixed-methods approach.

The aspire of this research is to bridge the gap by investigating the Land Use and Land Cover (LULC) changes and the influencing variables in the Gilgel Gibe basin between 1991 and 2021. It employs a combination of remote sensing, geographic information systems (GIS), and social survey approaches. In particular, this project seeks to accomplish the following objectives: (1) employ multi-temporal satellite images to scrutinize the extent, direction, and forms of Land Use and Land Cover (LULC) changes in the Gilgel Gibe basin; (2) Evaluate, utilizing data from statistical analysis and community opinion, the predominant causal agents behind the Land Use and Land Cover (LULC) transitions; and (3) Investigate the consequences of the Land Use and Land Cover (LULC) changes on the ecology and civilization of the Gilgel Gibe basin. This research play a part to advancing scientific understanding and policymaking regarding Land Use and Land Cover (LULC) movements and their impacts in Ethiopia at large, with a specific focus on the Gilgel Gibe basin.

## 2 Exploration zone and methodological approach

### 2.1 Unveiling the study locale

#### 2.1.1 Geographical coordinates

**Fig. 1:**
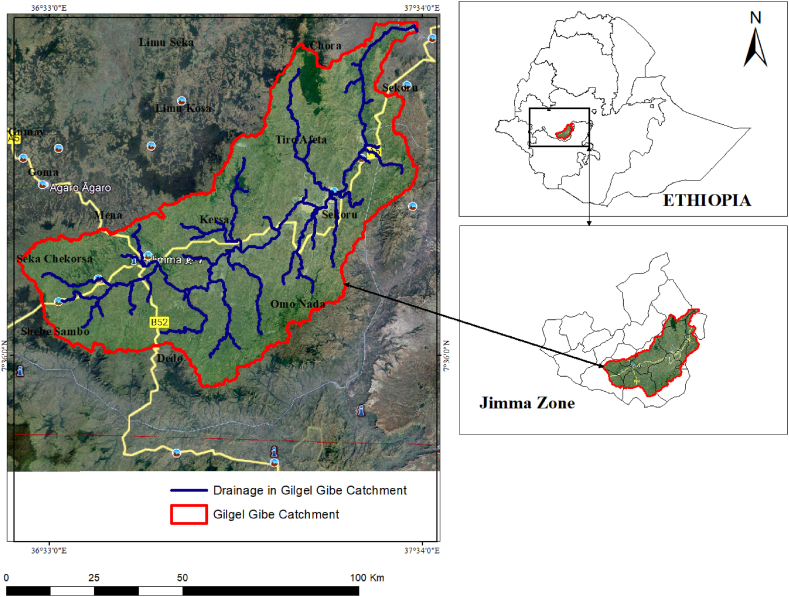
A map illustrating the study area.

The research has been carried out within the confines of the Gilgel Gibe Catchment, positioned upstream in the expansive Omo Gibe basin, nestled within the Jimma zone of the Oromia National Regional State in the southwestern region of Ethiopia. The site is situated 260 km southwest of Addis Ababa, with approximate geographic coordinates ranging from 7°36′0″ to 8°0′0″ N latitude and 36°36′0″ to 37°34′0″ E longitude (ArcGIS 10.5 Fig. 1). The watershed encompasses a region spanning approximately 514,103.4 ha, featuring elevations ranging from 1096 to 3341 m. a.s.l. The basin encompasses Sekoru, Tiro Afeta, Kersa, Seka Chekorsa, Omo Nada, Dedo, Shebe Sembo, and Chora districts within the Jimma zone and Jimma special town. The principal river in the catchment is the Gilgel Gibe, which intersects with the Gilgel Gibe hydroelectric dam [21].

Source [21]:

#### 2.1.2 Weather patterns

**Fig. 2:**
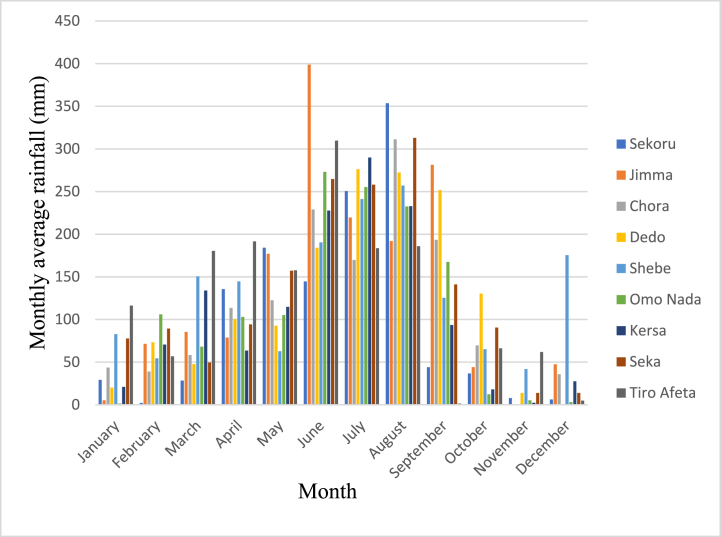
Illustrates the monthly average rainfall at stations within the Gilgel Gibe catchment.

**Fig. 3:**
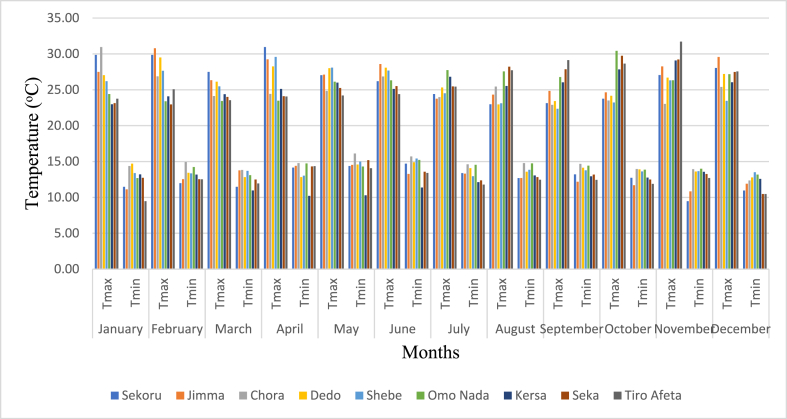
Displays the monthly extremes in temperature, both maximum and minimum, at stations situated within the Gilgel Gibe catchment.

Despite its tropical location, Ethiopia experiences a diverse array of climate fluctuations shaped by factors such as the Inter-Tropical Convergence Zone (ITCZ), altitude, and the presence of the rift valley. The nation witnesses average temperatures spanning below 10 °C to surpassing 40 °C, showcasing significant variations in both temperature and precipitation patterns. Annual precipitation levels range from 100 mm to 2800 mm (refer to Fig. 2), varying based on geographical location. Furthermore, rainfall distribution exhibits diversity, with certain regions experiencing two distinct rainy seasons (Belg from March to May and Kiremt from June to August), while others encounter a single rainy season [21]. The average temperature within the region ranges from 11.5 °C to 27.5 °C (refer to Fig. 3), accompanied by an average precipitation of 1521 mm. Notably, the basin's lower reaches contribute to these climatic variations.

Source [21]:

Source [21]:

#### 2.1.3 Description of LULC classes

Drawing upon prior understanding of the study region, insights gleaned from earlier research, direct on-site observations, and interviews with relevant local authorities, the Gilgel Gibe watershed has been categorized into seven distinct forms of land use and land cover. These are built up area cultivated land, forest, shrubland, degraded land, grazing land/grasslands and water bodies (Table 1).

**Table 1:** Definition of LULC classes

LULC Type	Definition about the LULC Type
Built-up area:	Structures like residences, commercial zones, public facilities, and infrastructure, including roads and parks, all developed by human activity, contribute to the built environment. This encompasses concrete and asphalt surfaces, marketplaces, institutions like schools and clinics, as well as dispersed rural homestead buildings forming clustered settlements.
Cultivated land:	Land designated for agriculture offers a range of uses, including planned plots, fields of crops irrigated or rain-fed, fallow areas, and land sporadically adorned with vegetation. In the context of agroforestry, this involves scattered chat plantations and annual crops.
Forest:	Locations characterized by the prevalence of trees, standing at heights ranging from two to 5 m, arranged in closely spaced stands with interlocking or nearly closed canopies, are widespread. These areas exhibit a distinct feature, namely the predominance of plantation woods, particularly featuring eucalyptus, grevillea robusta, and cupressus lusitanica.
Shrub-land:	Terrain adorned with diminutive trees, shrubs, and bushes, occasionally intermingled with grasses, displaying a density lower than that of traditional forests.
Degraded land:	Areas primarily consisting of exposed rocks and soil as a result of erosion, overgrazing, or deforestation comprise moderately steep and gently sloped mountain and hill sides.
Grazing-land	The terrain is predominantly adorned with native grasses and small shrubs, accompanied by herbaceous plants primarily utilized for animal and cattle grazing. In this context, the term “land cover” is employed expansively, encompassing a diverse array of vegetation, including legumes, grasses, various forbs, and sporadically, woody plants.
Water body	Terrain encompassing a petite reservoir replenished by water from streams and rainfall, along with sections collecting water from diverse sources such as puddles, wetlands, rivers, streams, and similar features.

### 2.2 Methodology employed for the research

#### 2.2.1 Sample size determination and data collection methods

Enhancing the precision and reliability of the classification involved fieldwork within the Gilgel Gibe watershed, incorporating the use of GPS and on-site observations. Beyond validating the accuracy of the mapped data, the study aimed to assess the physical characteristics of the watershed and elements associated with land use. The guidelines provided by Ref. [38] were adhered to when choosing training and accuracy assessment data for each Land Use/Land Cover (LU/LC) class. The total number of training samples was 350, 50 for each of the seven LU/LC classes. The accuracy assessment data were different from the training data and consisted of 458 samples, which were also randomly distributed among the LU/LC classes. The creation of the LULC map involved the application of picture enhancement, visual interpretation techniques, training and reference data, and supervised image classification, independent of the training set.

For the interview method, the sample size was 30, which corresponded to the number of respondents that were selected from different categories of stakeholders who were involved in or affected by the LU/LC changes in Gilgel Gibe catchment. The sample size was based on the purposive sampling strategy that aimed to achieve a balanced and diverse representation of the different categories of stakeholders, such as farmers, elders, guards of the forests, community leaders, extension workers (Development agents), and village administrators. Conversations with key informants provided insights into the watershed. Interviews were carried out with elderly residents, longtime locals, and forest rangers responsible for protected areas. The primary objective of these interviews was to gain a deeper understanding of historical and current trends in land use and conservation (LULC), along with the factors that have shaped these transformations.

For the focus group discussion method, the sample size was 60, which corresponded to the number of respondents that were divided into 10 groups of 6 participants each. The sample size was based on the purposive sampling strategy that aimed to create homogeneous and interactive groups of respondents who shared similar characteristics and experiences related to the LU/LC changes in Gilgel Gibe catchment.

For the Likert scale questionnaire method, the sample size was 90, which corresponded to the total number of respondents that participated in the interview and focus group discussion methods. The sample size was based on the purposive sampling strategy that aimed to ensure a sufficient and representative sample size for the Likert scale questionnaire data analysis, and to test the link amongst the LU/LC change index and the Likert scale scores [6,7,16,[39], [40], [41]] of the statements using regression analysis. The Likert scale questionnaire consisted of 20 statements that measured the respondents' perceptions and attitudes towards the LU/LC changes in Gilgel Gibe catchment. Participants utilized a five-point rating system, spanning from strongly disagree to strongly agree, to express their sentiments regarding each statement. In the context of the regression analysis, the independent variable employed was the LU/LC change index, while the dependent variable was represented by Likert scale scores. A formula incorporating both the area and percentage change for every land use/land cover category was conducted to calculate the Land Use/Land Cover (LU/LC) change index for the Gilgel Gibe watershed. The regression analysis aimed to examine whether there was a significant linear association amongst the LU/LC change index and the Likert scale scores, and to estimate regression line's slope and intercept.

#### 2.2.2 Methods for data analysis

##### 2.2.2.1 The processing of images

The examination of Land Use and Land Cover (LULC) changes in this study utilized Landsat TM imagery from 1991, ETM + photos from 2006, and Operational Land Imager (OLI) images from 2021, obtained through the United States Geological Survey (USGS) data portal at http://earthexplorer.usgs.gov. Data collection dates were deliberately chosen to align with the same season each year, with the aim of minimizing the influence of seasonal variations on vegetation patterns and distribution throughout the annual cycle. The identification of images involved referencing the Landsat grid, where specific path (p) and row (r) parameters were specified. Subsequently, Landsat images encompassing the entire watershed were acquired through two distinct paths and rows, as detailed in Table 2.

**Table 2:** Characteristics of images

Sensor Type	WRS (Path/Row)	Spatial Resolution (m)	Acquisition Date	Producer
TM	169_054	30 × 30	01-01-1991	USGS
TM	169**_**055	30 × 30	01-01-1991	USGS
TM	170_055	30 × 30	01-01-1991	USGS
ETM+	169_054	30 × 30	01-01-2006	USGS
ETM+	169_055	30 × 30	01-01-2006	USGS
ETM+	170_055	30 × 30	01-01-2006	USGS
OLI	169_054	30 × 30	01-01-2021	USGS
OLI	169_055	30 × 30	01-01-2021	USGS
OLI	170_055	30 × 30	01-01-2021	USGS

The thermal bands (band 6) from TM, ETM+, and OLI were excluded from this analysis because of their limited spatial resolution (120 and 60 m). After completing the pre-processing procedures, we amalgamated distinct satellite images using a distinctive standard RGB “false color composition (FCC)" specifically tailored for the satellite imagery of the research area. This stage aimed to enhance the clarity of observing, distinguishing, and interpreting surface features, thereby facilitating efficient classification within the study domain. The selection of band combinations was determined by their effectiveness in identifying features within the study area. Each band corresponds to a distinct set of data files within a specific range of the electromagnetic spectrum.

#### 2.2.3 Image classification

This research utilized Landsat data spanning three distinct years: Enhanced Thematic Mapper Plus data from 2006, Thematic Mapper data from 1991, and Operational Land Imager or Thermal Infrared Sensor data from 2021 for the classification of Land Use and Land Cover (LULC). Supervised classification, employing the maximum likelihood algorithm, was utilized to categorize and analyze these images. The study region was segmented into categories such as built-up areas, water bodies, degraded lands, forests, shrublands, and cultivated land within the framework of land-use and land-cover (LULC). In classification schemes and change detection analysis of satellite images 1991–2006, 2006–2021, and 1991–2021 were analyzed by using ENVI5.1 software in application of supervised categorization by using Maximum likelihood (MLH) categorizer algorithm. The maximum likelihood classifier, a parametric approach to image classification, assigns each pixel to the class with the highest probability in the resulting posterior probability distribution, determining its class membership [17,18,42,43]. The classification of Land Use and Land Cover (LULC) classes in the Gilgel Gibe Catchment employed the greatest likelihood approach [19,42].

**Fig. 4:**
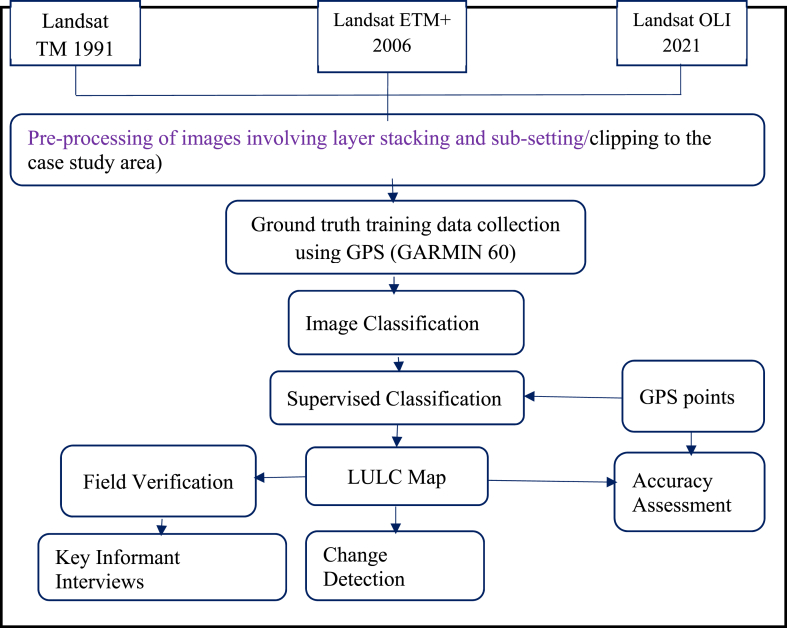
Flow diagram displaying the general approach used to classify land use and land cover (LULC).

The assessment involved the calculation of both the extent and shifts in each Land Use and Land Cover (LULC) category during the designated study periods. Lastly, using an ArcGIS overlay analysis technique, transformation matrices for the three periods (1991–2006, 2006–2021, and 1991–2021) were analyzed among the three consecutive maps depicting Land Use and Land Cover (LULC) of the catchment (Fig. 4). ArcGIS 10.5 was used for image processing, categorization, and analysis.

#### 2.2.4 Accuracy assessment techniques

A total of 428 field-collected ground truth points, chosen at random, were employed to assess the precision of the categorized photographs. These points were cross-referenced with Google Earth. Subsequent confusion matrix analysis followed the procedures outlined by Ref. [38].

The accuracy evaluation of the study area relied on the utilization of the confusion (error) matrix generated from the classified Land Use and Land Cover (LULC) data. The classification of Land Use and Land Cover (LULC) remains incomplete until the accuracy level reaches a satisfactory threshold, as per the criteria outlined by Ref. [38]. In this analysis, Kappa statistics are employed for evaluation, alongside the assessment of overall accuracy. The accuracy measurement involved collecting ground control points for each Land Use and Land Cover (LULC) class through the Garmin60 Global Positioning System (GPS). Google Earth was also used to acquire additional ground control points [38]. As per the findings of [24,[44], [45], [46], [47]], the Kappa coefficient (Khat) serves as a metric for assessing agreement between two maps, considering all elements within the confusion matrix.

#### 2.2.5 LULC change rate analysis

Rates and magnitudes of the LULC changes of Gilgel Gibe Catchment were quantified based on results (areas) from the GIS and Landsat images-based classification of seven (7) LULC classes during the three periods (1991, 2006 and 2021) accounted in by the study. The computation involved assessing the rate of Land Use and Land Cover (LULC) change for the intervals 1991–2006, 2006–2021, and 1991–2021. To illustrate the extent of changes during specified intervals, the magnitude of change was determined using the calculation outlined in Equation (1).

(1) $$Rate\;of\;change\;\left(\frac{ha}{year}\right)=\frac{A2\;-\;A1}{Z}$$

In the context of Equation (2) (Eq. (2)), A2 represents the area of a specific LULC class in hectares (ha) during year 2, A1 signifies the corresponding area (ha) of the same LULC class in year 1, and Z indicates the time span between A2 and A1 in years. Based on the studies conducted by Refs. [24,41,48,49], computations were undertaken to determine the extents (measured in hectares) and proportions of changes within Land Use and Land Cover (LULC) categories over the intervals 1991–2006, 2006–2021, and 1991–2021.

(2) $$\text{Percent}\;\left(\%\right)\;\text{of}\;\text{LULC}\;\text{change}=\frac{Area\;at\;final\;year-Area\;at\;initial\;year}{Area\;at\;initial\;year}*100$$

Finally, analysis results about the rates (per/year) and magnitudes (for 15 and 30 years-intervals) of changes of the LULC classes were organized, illustrated and presented in tabular and figure (map, line-graph and bar-graph) forms. The findings of the study were interpreted. Evidences of field observation and key informants were used to cross-check with results of the image interpretation and classification of the LULC classes of Gilgel Gibe Catchment, and also to strengthen the interpretation of study results. Discussion was also made in comparison with evidences of field observation and results of studies made on related issues.

#### 2.2.6 Data analysis technique for interviews, FGD and likert scale questionnaire

Qualitative data analysis and statistical analysis of numerical data. The analysis of qualitative data was carried out concerning data collected from the interview and focus group discussion methods, while the quantitative data analysis was conducted on the information gathered from Likert scale questionnaire method [11,23,30,40,[50], [51], [52]]. The following steps were followed for each part pertaining to the data analysis:

**Analysis of qualitative data:** Involved transcribing interviews and focus group discussions into written texts. The texts were analyzed according to the themes and sub-themes related to the research questions. The themes and sub-themes derived from participants' reports encompass a range of observable alterations in land use and land cover, exploring the underlying mechanisms influencing these changes. The implications of these transformations on livelihoods and the environment, among other factors, were also incorporated into the identified themes. The themes and sub-themes were described and explained in relation to the research questions. Direct quotes from the respondents were used to illustrate the points.

**Quantitative data analysis:** The Likert scale scores for each statement and each category of respondents were entered into a statistical software (SPSS) and analyzed using regression analysis. Regression examination was conducted to scrutinize test the link in the midst LU/LC change index and the Likert scale scores of the statements. The dependent variable in this study comprised Likert scale ratings, with the independent variable being the LU/LC change index. The intercept and slope of the regression line were identified. The coefficient of determination (R-squared) and the p-value were calculated to assess the correlation's significance and strength. The results of both qualitative and quantitative data analysis were integrated and compared at the interpretation stage. The findings were discussed in relation to the literature review and the theoretical framework.

## 3 Results and discussion

### 3.1 Verification of accuracy

In this research, an assessment was conducted to prove whether results of the satellite images’-based classifications of LULC classes of Gilgel Gibe Catchment were accurate in comparison with the realities in the catchment.

Findings from the Accuracy evaluation about the LULC classes of the catchment revealed that the overall classification accuracies were 86.5 %, 94.4 %, and 85.1 % for the period 1991, 2006 and 2021, respectively. These accuracy values imply that 86.5 % of the 1991 LULC classes, 94.4 % of the 2006 LULC classes and 85.1 % of the 2021 land use and land cover categories within the research region were accurately classified. The overall Kappa statistics of the LULC classes for the period 1991, 2006 and 2021 were 0.91, 0.95 and 0.89, respectively (Table 3). The Users' Accuracies (UA) of individual LULC classes range from 85.2 % of ‘grazing-land’ (in 2006) to 100 % for ‘built-up area’ (in 2021).

**Table 3:** Producers' accuracy (PA) and users’ accuracy (UA) (%) of LULC classes for 1991–2021

No	LULC Classes	1991		2006		2021	
		PA (%)	UA (%)	PA (%)	UA (%)	PA (%)	UA (%)
1	Built-up area	100.0	96.6	100.0	99.8	100.0	100.0
2	Cultivated land	86.1	87.9	88.5	98.9	86.9	97.0
3	Forest cover	94.1	92.3	88.9	94.1	100.0	86.1
4	Shrubland	95.4	86.3	91.8	89.6	93.1	91.4
5	Degraded land	88.1	87.2	91.5	89.1	98.0	91.0
6	Grazing-land	93.8	89.1	89.8	85.2	100.0	87.3
7	Water body	99.1	97.2	100.0	99.9	99.8	99.5
8	Overall Accuracy	86.5	–	94.4	–	85.1	–
9	Kappa Coefficient	0.91	–	0.95	–	0.89	–

The Producers’ Accuracies (PA) of LULC classes range from 86.1 % of cultivated land (in 1991) to 100 % of grazing-land (2021), built-up area (1991, 2006 and 2021) and forest (2021) (Table 3). The overall accuracies pertaining to the classified land use and land cover (LULC) categories derived from Landsat imagery of Gilgel Gibe Catchment were larger than the minimum standard level (85 %) of accuracy for effective LULC classification and change detection [6,[53], [54], [55], [56], [57]].

### 3.2 Conversion of Gilgel Gibe Catchment's land cover and land use

Table 4 illustrates the primary changes in LULC within the examined region.

**Table 4:** LULC matrix of the study area

		Area of LULC of 2021 (ha)							
		Built-up area	Cultivated land	Forest	Shrubland	Degraded land	Grazing-land	Water body	Total
**Area of LULC 1991 (ha)**	Built-up area	10,381.63	1253	112.7	25.03	11.02	29.02	0	6676.81
	Cultivated land	2386.39	58,821.6	418.83	92.84	188.67	16,008.98	26.03	277,560.50
	Forest	1382.59	123,919.71	19,021.3	179.13	26.53	29,848.87	154.45	52,113.09
	Shrubland	998.14	49,854.96	4501.43	551.9	21.73	31,777.45	184.53	1727.93
	Degraded land	883.96	43,969.69	5007.86	13.02	192.14	14.01	345.15	275.06
	Grazing-land	1025.63	58,437.84	4029.50	287.06	12.05	47,084.2	598.04	172,099.84
	Water body	0.1	125.3	0.2	27.05	15.06	253.04	2738.04	3650.17
	Total	17,058.44	336,382.10	33,091.83	1176.03	467.20	125,015.67	912.13	51,4103.4

The diagonal in Table 4 indicates the Land Use and Land Cover (LULC) class that remains consistent from 1991 to 2021. Simultaneously, the sum along the vertical axis represents the cumulative LULC gains from the initial year to the final year, while the total along the horizontal axis of the table signifies the starting year of LULC losses. Based on the findings, there was an escalation in the built-up area and cultivated land at the expense of the forest, with gains of 1382.59 ha and 123,919.71 ha, respectively, between 1991 and 2021. Similarly, the built-up area and cultivated land expanded at the cost of grazing land, showing increments of 1025.63 ha and 58,437.84 ha, respectively, over the same period. The report indicates that from 1991 to 2021, there was an expansion of cultivated land and built-up areas encroaching upon shrubland, grazing land, and forest. These findings align with research conducted by Refs. [18,23,49,58]. According to the study, there has been a significant conversion of grassland and forest to built-up areas and cultivated land.

### 3.3 The LULC classes of Gilgel Gibe Catchment

LULC categories within the research zone were determined through the analysis of Landsat images captured in 1991, 2006, and 2021. Result of the interpretation Landsat images revealed that cultivated land, grazing land/grasslands, built up area, shrubland, forest, water body and degraded land were the main LULC classes of Gilgel Gibe Catchment (Table 4 and Fig. 3).

**Fig. 5:**
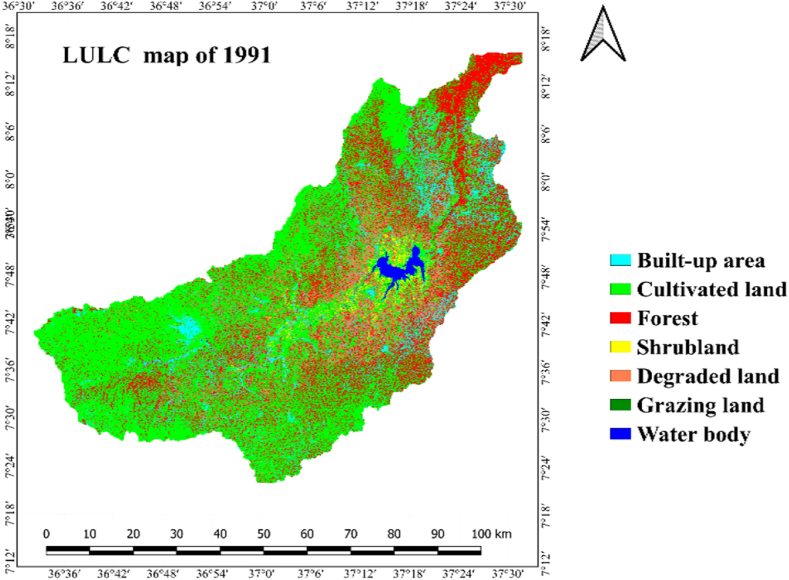
Lulc classes of gilgel gibe catchment in 1991.

Based on the findings of the investigation, cultivated land with the respective area proportion of 54 % (277560.50 ha), 57.9 % (297691.3 ha) and 65.4 % (336382.10 ha) was the most dominated LULC class during all the three periods (i.e.1991, 2006 and 2021) accounted in by the study (Table 4; Fig. 5). The greatest share (87.5 % or 449660.3 ha) from the whole catchment area in 1991 was accounted by cultivated land and grazing-land. In 1991, forest and built-up area cover accounted 10.1 % (52113.1 ha) and 1.3 % (6676.8 ha), respectively. The share of water body (0.7 % or 3650.2 ha), shrubland (0.3 % or 1727.93 ha) and degraded land (0.1 % or 275.1 ha) in the catchment's overall area was insignificant in the period 1991 (Table 4; Fig. 5).

Cultivated land and grazing-land, with the respective area of 57.9 % (297,691.3 ha) and 31.3 % (161,016.8 ha), constituted the largest proportion (89.2 %) of Gilgel Gibe Catchment in 2006. Forest and built-up area covers, with the respective share of 7.7 % (39,780.7 ha) and 2.2 % (11,316 ha) of the study area, were the third and fourth LULC classes in 2006. The 2006 area of water body (0.4 %), shrub-land (0.3 %) and degraded land (0.1 %) was insignificant.

As it is illustrated in Table 4 and Fig. 5, cultivated land and grazing land class cover the widespread share of LULC classes, which account an area of 89.8 % (9461397.8 ha). Forest and built-up area an aerial size of 6.4 % (933091.8 ha) and 3.32 % (17058.44 ha), respectively. Shrubland and water body cover 0.23 % (1176.03 ha) and 0.18 % (912.13 ha), respectively. The smallest areal coverage is degraded land, which accounts for only 0.09 % (467.2 ha) from the whole of the catchment. As per the data presented in Table 4 and depicted in Fig. 5, it can be inferred that, in the year 2021, cultivated land and grazing land constituted 89.8 % of the entire catchment area. The remaining 10.3 % encompassed forest, built areas, shrubland, water bodies, and degraded land.

### 3.4 Magnitude and trend of LULC dynamics in Gilgel Gibe Catchment in 1991–2021

**Fig. 8:**
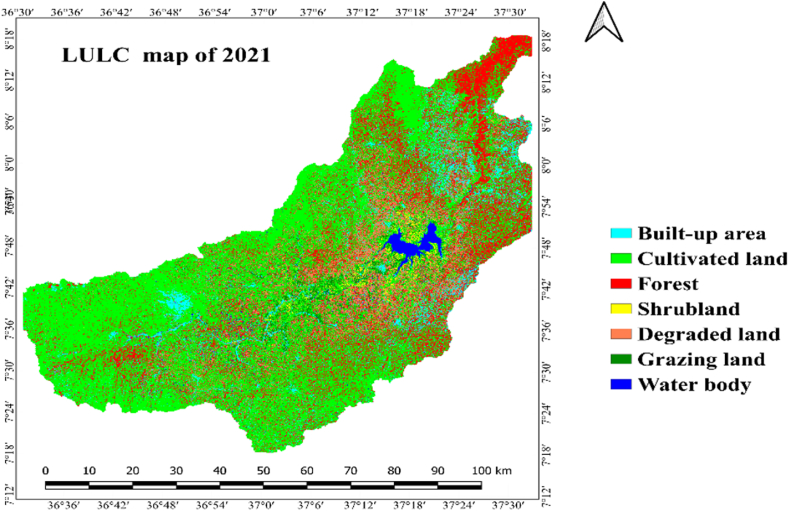
Lulc classes of gilgel gibe catchment in 2021.

The study attempted to quantify the magnitudes of changes of the seven LULC classes of Gilgel Gibe Catchment for 1991–2006, 2006–2021 and 1991–2021. Besides, an assessment was also conducted so as to prove whether the LULC classes of the catchment revealed clear patterns in their trends of change in thirty years (1991–2021). Results of these issues can be observed in Fig. 7 and Table 5Fig. 8.

**Table 5:** Area (A) and percent (%) of LULC classes of gilgel gibe catchment in 1991, 2006 and 2021

N0	LULC Classes	1991		2006		2021	
		Area (ha)	P (%)	Area (ha)	P (%)	Area (ha)	P (%)
1	Built-up area	6676.8	1.3	11,316.0	2.2	17,058.4	3.3
2	Cultivated land	277,560.5	54.0	297,691.3	57.9	336,382.1	65.4
3	Forest cover	52,113.1	10.1	39,780.7	7.7	33,091.8	6.4
4	Shrubland	1727.9	0.3	1601.5	0.3	1176.0	0.2
5	Degraded land	275.1	0.1	554.1	0.1	467.2	0.1
6	Grazing-land	172,099.8	33.5	161,016.8	31.3	125,015.7	24.3
7	Water body	3650.2	0.7	2143.1	0.4	912.1	0.2
	Total	514,103.4	100.0	514,103.4	100.0	514,103.4	100.0

**Fig. 6:**
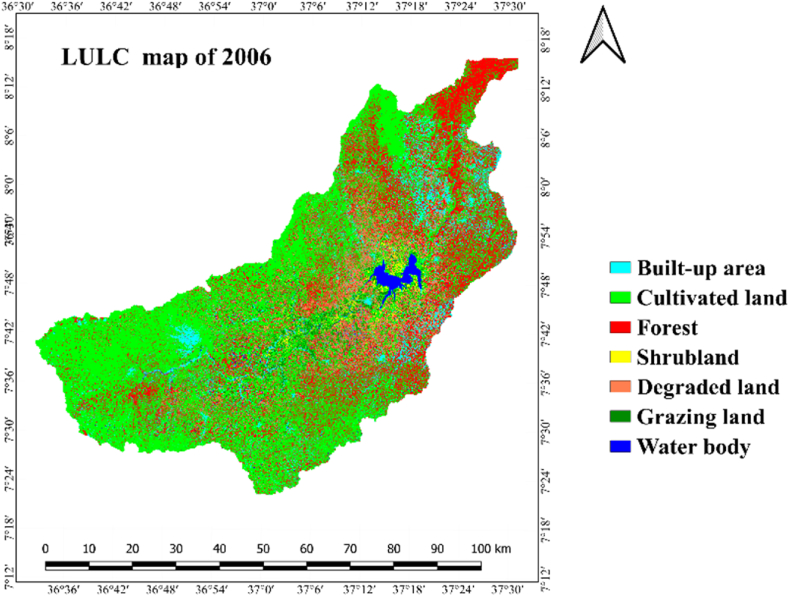
Lulc classes of gilgel gibe catchment in 2006.

As it is clearly displayed within Fig. 6 and Table 5, the expanse designated as cropland or cultivated land of Gilgel Gibe catchment had increased by 7.3 % (20,130.8 ha) in 1991–2006 and, it also increased by 13 % (38,690.8 ha) in 2006–2021. Similarly, built-up area had expanded by 69.5 % (4639.2 ha) and 50.7 % (5742.4 ha) in the period 1991–2006 and 2006–2021, respectively. Said another way, built-up area and cultivated land of the study catchment were expanding with increasing magnitude of change in the last thirty years (1991–2021); and, these results clearly reveal that built-up area and cultivated land have been continuously increasing in the 30 years-period (1991–2021) studied. Agriculture and settlement expansion, and over-exploitation of forest and shrub resources were among the main driving forces for the increment in built-up area and cultivated land in the catchment.

**Fig. 7:**
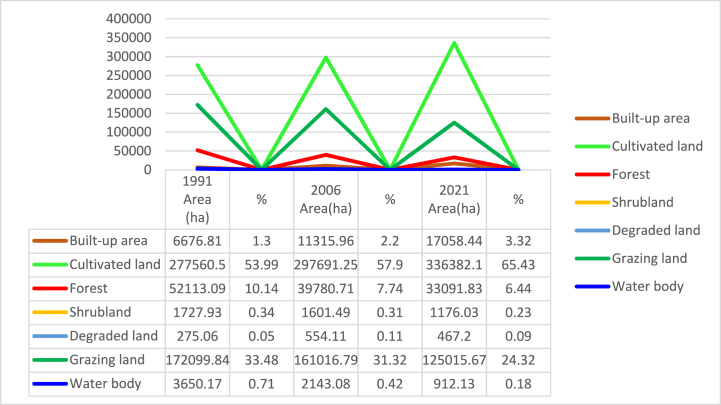
Area share (%) of LULC classes of gilgel gibe catchment in 1991, 2006 and 2021.

On the contrary, forest cover of Gilgel Gibe Catchment had declined by 23.7 % (12,332.4 ha) and 16.8 % (6688.9 ha) in 1991–2006 and 2006–2021, respectively. In similar fashion, water body cover of the catchment was decreasing by 41.3 % (1507.1 ha) and 57.4 % (1231 ha) in 1991–2006 and 2006–2021, respectively (Table 5). While grazing land and shrubland declined by the respective magnitude of change of 6.4 % (11,083 ha) and 7.3 % (126.4 ha) in the period 1991–2006, the same land cover classes continued to decline by 22.4 % (36,001.1 ha) and 26.6 % (425.5 ha) in the last fifteen years (2006–2021), respectively (Table 5). These findings imply that forest cover and water body of Gilgel Gibe Catchment were declining with decreasing magnitudes of changes in the three-decades (1991–2021) studied; whereas, grazing land and shrubland covers were shrinking with increasing magnitude of change in the last thirty years (1991–2021) (Table 5 and Fig. 7). Evidences of interviewees and own field observation confirm that the rapid population increase and the subsequent increasing demand for farmland and residential area in Gilgel Gibe Catchment has led to the dramatic shrinkage of forest, grazing land, water body and shrubland resources in the catchment.

**Fig. 9:**
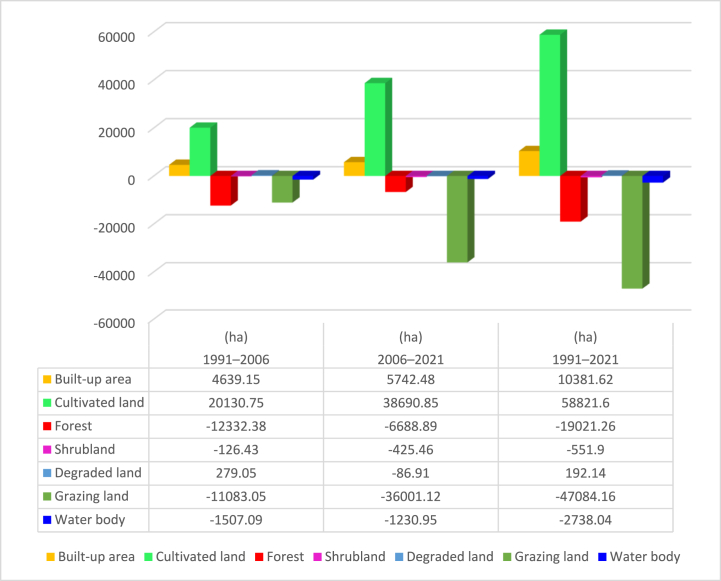
Changes of the LULC classes of gilgel gibe catchment in 1991–2021.

Degraded (bare) land is the other LULC class that has been accounted in the assessment of the LULC dynamics in Gilgel Gibe Catchment. The study's findings indicated that deteriorated land/degraded land of the catchment has risen by two-fold (101.4 % or 279 ha) in the first fifteen years (1991–2006) accounted in by the study. However, area coverage of the degraded land of the catchment declined by 15.7 % (86.9 ha) in the last fifteen years (2006–2021) studied (Table 5). Similar to the results of this research, the result of another study made on LULC conversions (at northwestern Ethiopia) revealed that the extent of degraded land was increasing overtime [7,57,[59], [60], [61], [62], [63], [64], [65], [66], [67], [68]] (see Fig. 9).

**Fig. 10:**
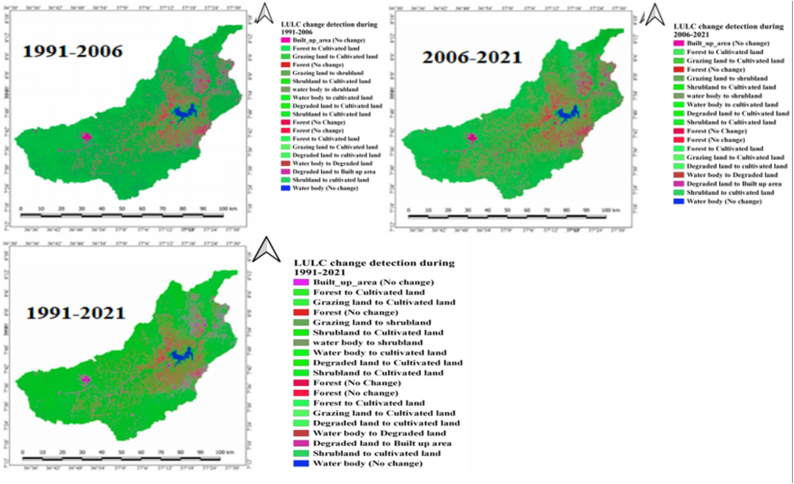
Pristine natural areas that have shown no alterations in land utilization and cover transformations since 1991–2021.

On the other hand, the magnitudes (gains and losses) of dynamics of the LULC classes of Gilgel Gibe Catchment were computed for thirty years (1991–2021). The results of the study revealed that Gilgel Gibe catchment had experienced net gains (increases) in built-up area by 155.5 % (10,381.6 ha) and in cultivated land by 21.2 % (58,821.6 ha) in the three-decades studied. However, grazing land forest covers exhibited net shrinkage (loss) by the respective magnitudes of 27.4 % (47,084.1 ha) and 36.5 % (19,021.3 ha) in 30 years. Similarly, water body and shrubland covers of the catchment also experienced net decline (loss) by 75 % (2738.1 ha) and 31.9 % (551.9 ha), respectively, in the same period. Even if degraded land of Gilgel Gibe catchment exhibited a declining trend in the last fifteen years (2006–2021), this LULC class experienced a net increase by 69.8 % (192.1 ha) in three decades (1991–2021) considered by the study (Fig. 10). Thus, the net rise in cultivated land, and built-up area, degraded land versus the net loss (decline) in forest, shrubland, grazing land and water body lead us to the implication that rapid population growth-induced anthropogenic activities have been the main catalysts for the dynamics of Land Use and Land Cover (LULC), depletion of environmental resource (e.g. forest, shrubland, grassland and water) and increasing degradation of Gilgel Gibe Catchment in Southern Ethiopia.

The results of analysis utilizing remote sensing and GIS techniques were consistent with the findings of other studies that have analyzed LU/LC changes in Ethiopia and other regions using similar methods and data sources. For example [69], used Landsat images from 1986 to 2015 to classify the LU/LC types and calculate the percentage and area of change for each LULC type in Upper Blue Nile Basin, Ethiopia. They found a significant decrease of grassland and, woodland, and a significant increase of built-up areas and agricultural land in the basin. They also found that there were spatial variations and heterogeneities in the LU/LC changes across different sub-basins in the basin. Similarly [70,71], used Landsat images from 1990 to 2015 to classify the LU/LC types and calculate the percentage and area of change for each type in Chitwan-Annapurna Landscape, Nepal. They found that there was a significant decrease of forest and water, and notable rise of cropland and settlements of the landscape. They also found that there were spatial variations and heterogeneities in the LU/LC changes across different districts in the landscape.

### 3.5 Annual rate of LULC changes of Gilgel Gibe Catchment in 1991–2021

Annual rates of changes have been calculated about the seven (7) LULC classes of Gilgel Gibe Catchment for 30 years (1991–2021) just so as to understand the rate of gain and/or loss of each LULC class per/year (Table 6 below) (see Table 7).

**Table 6:** Area (A) and percent (%) of change of LULC classes in 1991–2006, 2006–2021 and 1991–2021

N0	LULC	1991	2006	2021	1991–2006		2006–2021		1991–2021	
	A (ha)	A (ha)	A (ha)	A (ha)	A (ha)	%	A (ha)	%	A (ha)	%
1	Built-up area	6676.8	11,316.0	17,058.4	4639.2	69.5	5742.4	50.7	10381.6	155.5
2	Cultivated land	277,560.5	297,691.3	336,382.1	20130.8	7.3	38690.8	13.0	58821.6	21.2
3	Forest cover	52,113.1	39,780.7	33,091.8	−12332.4	−23.7	−6688.9	−16.8	−19021.3	−36.5
4	Shrubland	1727.9	1601.5	1176.0	−126.4	−7.3	−425.5	−26.6	−551.9	−31.9
5	Degraded land	275.1	554.1	467.2	279.0	101.4	−86.9	−15.7	192.1	69.8
6	Grazing land	172,099.8	161,016.8	125,015.7	−11083	−6.4	−36001.1	−22.4	−47084.1	−27.4
7	Water body	3650.2	2143.1	912.1	−1507.1	−41.3	−1231.0	−57.4	−2738.1	−75.0
	**Total**	**514,103.4**	**514,103.4**	**514,103.4**	**0**	**0.0**	**0**	**0.0**	**0**	**0.0**

**Table 7:** Annual rate (R) (%) of LULC changes in 1991–2006, 2006–2021 and 1991–2021

N0	LULC	1991	2006	2021	1991–2006		2006–2021		1991–2021	
	A (ha)	A (ha)	A (ha)	A (ha)	R (ha)	%	R (ha)	%	R (ha)	%
1	Built-up area	6676.8	11,316.0	17,058.4	309.3	4.6	382.8	3.4	346.1	5.2
2	Cultivated	277,560.5	297,691.3	336,382.1	1342.1	0.5	2579.4	0.9	1960.7	0.7
3	Forest	52,113.1	39,780.7	33,091.8	−822.2	−1.6	−445.9	−1.1	−634.0	−1.2
4	Shrub-land	1727.9	1601.5	1176.0	−8.4	−0.5	−28.4	−1.8	−18.4	−1.1
5	Degraded	275.1	554.1	467.2	18.6	6.8	−5.8	−1.0	6.4	2.3
6	Grazing land	172,099.8	161,016.8	125,015.7	−738.9	−0.4	−2400.1	−1.5	−1569.5	−0.9
7	Water body	3650.2	2143.1	912.1	−100.5	−2.8	−82.1	−3.8	−91.3	−2.5
	**Total**	**514,103.4**	**514,103.4**	**514,103.4**	**0**	**0.0**	**0**	**0.0**	**0**	**0.0**

Agricultural/Cultivated land and built-up area had increased by the respective rates of 0.5 % (1342.1 ha) and 4.6 % (309.3 ha) per/year in the first 15 years (1991–2006), and by 0.9 % (2579.4 ha) and 3.4 % (382.8 ha) per/year in the last 15 years (2006–2021). On average, built-up area and cultivated land of Gilgel Gibe Catchment were increasing by the respective rates of 5.2 % (346.1 ha) and 0.7 % (1960.7 ha) per year in three decades (1991–2021) (Table 6).

Forest cover of the catchment had declined by an annual rate of loss of 1.6 % (822.2 ha) in 1991–2006, and 1.1 % (445.8 ha) in 2006–2021. The annual rate of shrinkage of forest cover of the study area was 1.2 % (634 ha) in each of the 30 years (1991–2021) studied. Grazing land, water body and shrubland covers of Gilgel Gibe Catchment had continuously shrunk by annual rate of loss of 0.4 % (738.9 ha), 2.8 % (100.5 ha) and 0.5 % (8.4 ha) each year, respectively, in the first one and half decade (1991–2006). The annual rates of decline of grazing land and shrubland cover, with the respective magnitude of loss of 1.5 % (2400.1 ha) and 1.8 % (28.4 ha) in the last 15 years (2006–2021), were higher than the corresponding yearly depletion rate of the LULC classes in the initial period (1991–2006) studied. And, water cover declined at 3.8 % (82.1 ha) per/year within 2006–2021. While grazing land and shrubland covers of the catchment had declined by the respective annual rate of loss of 0.9 % (1569.5 ha) and 1.1 % (18.4 ha) per/year in three decades (1991–2021), water body had shrunk by 2.5 % (91.3 ha) per/year in the same period.

Again, degraded land of Gilgel Gibe catchment had increased by about 6.8 % (18.6 ha) each year in the initial period (1991–2006) studied; whereas, its area extent has declined by 1 % (5.8 ha) per year in the last one and half decade (2006–2021). However, the area of degraded (bare) land of the catchment was increasing experiencing an annual fluctuation rate of 2.3 % (6.4 ha) per/year in the last thirty years (1991–2021). The increasing degraded land is a reflection of the increasing degradation of environmental resources in Gilgel Gibe Catchment, Southern Ethiopia.

### 3.6 Drivers of the LULC dynamics of Gilgel Gibe Catchment in 1991–2021

Key informants were asked about the primary (perceived) catalysts behind the alterations in land use and land cover within Gilgel Gibe Catchment. According to the interviewees, rapid expansion of the population is the primary root (indirect) factor for the LU/LC dynamics and degradation of the study catchment. In fact, based on Ethiopia's 2007 demographic census, the total population of the Districts in Gilgel Gibe catchment such as Seka-Chekorsa (206,427), Sokoru (136,297), Kersa (164,053), Dedo (290,457), Omonada (246,008), Mana (149,661) and Shebe Sembo (112,267) was 1.3 million (1,305,170) [72]. The 1994 population of the study area was found to have grown by 27 % in 2007; and, 94.5 % of the 2007 people was residing in rural parts. Studies indicate that high population is the primary factor of LU/LC alterations in developing countries [14,35,45,53,[73], [74], [75], [76], [77], [78], [79], [80], [81], [82], [83], [84]].

On the basis of on responses of the key informants, factors such as scarcity of farmland, decline soil fertility and productivity, rainfall variability, firewood and charcoal production, unemployment, and open access to limited protection and low conservation of resources are the primary contributing factors of the LU/LC alteration in the catchment. An informant, for instance, stated that farmers of the area often tend to encroach to forest, shrub and grazing areas for acquisition of new farmland for their youth and/or for enhancing production of food crop that meet the needs of the growing family size. Another informant also pointed out that poor, landless and unemployed people produce firewood and charcoal from forest and shrub resources of the catchment (illegally) just to generate cash for their livelihoods.

The results of the remote sensing and GIS analysis also provided a basis for further analysis using other methods, such as interview, focus group discussion, Likert scale questionnaire, and regression analysis. These methods helped to identify and evaluate the main driving forces and consequences of LULC changes on the basis of the perceptions and experiences of the local community.

### 3.7 Qualitative analysis of stakeholders’ views on land use/land cover changes

The results presented herein stem from data acquired through focus groups and interviews involving ninety participants within the Gilgel Gibe Catchment. The participants were selected using purposive sampling to represent different stakeholders involved in or affected by the land use/land cover alteration, such as farmers, elders, guards of the forests, community leaders, extension workers (Development agents), and village administrators (local authorities). The data were analyzed using thematic analysis to identify the main themes and subthemes related to the influences and consequences of alterations in land use and land cover within the examined region.

The interviews were conducted with 30 participants who were elders and guards of the forests in the catchment. The interviews aimed to gain deep understanding on the past and present trends of LULC situations and the circumstances that are causing the transformations. The focus group discussions were conducted with 60 participants who were divided into 10 groups of six participants each. The focus group discussions aimed to elicit the perceptions and attitudes of the participants towards these changes.

#### 3.7.1 Population growth and LULC

The data analysis revealed that population growth was a major theme that influenced LULC changes in the catchment. The interviewees and focus group participants stated that the increasing population led to a higher demand for land for various purposes, such as agriculture, housing, and buildout. This led to the transformation of naturally occurring landscapes into different land use types, causing deforestation and urbanization. For instance, one of the interviewees said:

“The population is growing very fast here. There is not enough land for everyone. People need land for farming, for building houses, for roads, for schools, for hospitals. So they cut down the trees and clear the land. They change the natural environment into human-made environment.” (Interviewee 12, farmer)

This outcome aligns with similar research findings indicating that have shown that population growth is a key factor that influences LULC changes [12,33,65,80,[85], [86], [87], [88], [89], [90], [91], [92], [93], [94], [95]].

#### 3.7.2 Agricultural expansion and LULC

Another theme that emerged from the data analysis was the role of agricultural expansion as a driving force of LULC transformations in Gilgel Gibe Catchment. The interviewees and focus group participants reported that to meet the growing food needs, farmers cleared forests and natural habitats, creating new agricultural lands. This had negative ecological consequences, such as deforestation, soil erosion, and biodiversity loss. For example, one of the focus group participants said:

“We have to feed our families. We have to produce more food. So we have to expand our farms. We have to find new lands for cultivation. We have to clear the forests and the grasslands. We have to use fertilizers and pesticides. We have to irrigate our crops. But we don’t realize that we are destroying our environment.” (Focus group participant 7, farmer)

This outcome aligns with similar research findings indicating that have shown that agricultural expansion is another factor that drives LULC transformations in other catchments in Ethiopia, such as the higher reaches of the Awash Basin [96], the Jemma River Basin [97], and the Lake Ziway Watershed [98]. They have found that agricultural expansion occurs when farmers clear forests and other natural areas to create new agricultural land to fulfill the increasing need for food.

#### 3.7.3 Built-up area expansion and LULC

Additionally, our study identified built-up area expansion as a significant contributor to LULC conversion. The interviewees and focus group participants reported that this conversion was related to the simultaneous growth of population and the need for habitation and infrastructure. This caused the alteration of agricultural lands and forested areas into urban and industrial landscapes. For instance, one of the interviewees said:

“There is a lot of buildings going on here. There are new settlements, new roads, new dams. There is less green space, less nature, less wildlife.” (Interviewee 21, local authority)

This finding is similar to earlier researches that have demonstrated that the expansion of built environments is a significant contributor to LULC changes in other catchments in Ethiopia, such as the higher region of the Blue Nile Watershed [69], the Choke Mountain Range [96], and the Tana Lake Watershed [99]. They have ascertained that urbanization, rise in population, and cultivated land expansion are the main contributors of LU/LC alterations in these catchments, leading to deforestation, degradation of the land, erosion of the soil, quality of water deterioration.

#### 3.7.4 Deforestation and LULC

Our investigation also confirmed deforestation as a primary driving factor for land use and land cover fluctuations within Gilgel Gibe Catchment. The interviewees and focus group participants reported that the indiscriminate clearing of forests to accommodate agricultural and developmental activities provoked a cascade of ecological issues, like the erosion of soil, flooding, and climate change impacts. For example, one of the focus group participants said:

“The forests are disappearing here. They are being cut down for different reasons: for farming, for construction, for firewood, for charcoal, for timber. But they are not being replanted or protected. This causes many problems: the soil becomes loose and erodes away; the water becomes scarce and floods occur; the temperature becomes hotter and droughts happen.” (Focus group participant 15, local authority)

This finding is consistent with earlier researches indicating that deforestation is a major driving factor of LULC fluctuations in other catchments in Ethiopia, such as the Abay River Basin [66], the Bale Mountains [14], and the Lake Ziway Watershed [100]. They have found that deforestation is mainly caused by agricultural expansion, population growth, and fuelwood demand, and that it leads to soil erosion, water scarcity, and habitat loss.

#### 3.7.5 Land degradation and LULC

The data analysis also revealed the significant role of land degradation in causing LULC shifts. The interviewees and focus group participants reported that the irresponsible use and abuse of land reduced its productivity and increased its vulnerability to erosion and other threats. The resulting effects were a diminished agricultural resource base and a degraded ecosystem. For example, one of the interviewees said:

“The land is becoming degraded here. It is losing its fertility and quality. It is being overused and misused. People are not taking care of it. They are not using sustainable practices. They are not conserving the soil and water. They are not restoring the vegetation. This affects the productivity and profitability of the land. It also affects the health and well-being of the people and animals.” (Interviewee 27, local elder)

This result aligns with earlier researches that have shown that land degradation is an important factor that causes LULC shifts in other catchments in Ethiopia, such as the Awash River Basin [96], the Lakes Basin of Rift Valley [101], and the Tekeze-Atbara Basin [102]. They have found that land degradation is mainly driven by population pressure, agricultural expansion, overgrazing, and climate variability, and that it leads to soil erosion, sedimentation, nutrient loss, and biodiversity decline.

#### 3.7.6 Demand for farmland and forest products and LULC

The data analysis also emphasized the increased importance of the need for farmland and forest lands as a contributing force for LULC alterations. The interviewees and focus group participants reported that this demand increase, driven by population growth, stimulated a request for food and forest-based products. The ensuing outcome often involved the conversion of natural landscapes into agricultural domains. For example, one of the focus group participants said:

“The demand for land and forest products is increasing here. People want more food, more goods. They want more crops, more livestock. They also want more wood, more fuel. But they don’t have enough land or forest to meet their needs. So they change the natural landscape into agricultural or industrial landscape.” (Focus group participant 3, farmer)

This finding is similar to previous studies that have shown that the demand for farmland and forest products is a driving force for LULC changes in other catchments in Ethiopia, such as the higher reaches of the Awash Watershed [96], the Jemma River Basin [97], and the Lake Ziway Watershed [100]. They have found that the demand for farmland and forest products is driven by the population growth, income growth, and dietary changes, and that it leads to the alteration of natural areas to cultivation and industrial uses.

#### 3.7.7 Unemployment and LULC

Moreover, the data analysis revealed that unemployment was a significant catalyst for LULC alterations. The interviewees and focus group participants reported that when faced with unemployment, individuals may resort to forest clearing or other activities that accelerate LULC change, in pursuit of livelihoods or alternative opportunities. For example, one of the interviewees said:

“There is a lot of unemployment here. There are not enough jobs for everyone. People are poor and desperate. They have no other options. So they go to the forest and clear it for farming or other activities. They think they can make some money or survive somehow. But they don’t realize that they are harming themselves and their environment.” (Interviewee 9, farmer)

This result aligns with earlier researches that have shown that unemployment is a significant catalyst for LULC alterations in other catchments in Ethiopia, such as the Upper Tekeze Basin [102], the Lake Tana Basin [103], and the watershed of the Awash River [20]. They have found that unemployment is a factor that influences people to clear forests and other natural areas to create new agricultural land or to engage in other activities that can lead to LULC change.

#### 3.7.8 Lack of alternative income source and LULC

Our analysis also highlighted the paramount importance of alternative income sources in shaping LULC patterns. The interviewees and focus group participants reported that a lack of alternative means of livelihood may force individuals towards forest clearing and other similar actions to secure incomes, leading to further changes in the landscape. For example, one of the focus group participants said:

“There is a lack of alternative income sources here. People depend on farming or forest products for their income. They have no other skills or opportunities. They have no access to credit or markets. They have no support from the government or NGOs. So they have to rely on what they have: land and forest. But they use them unsustainably and inefficiently. They change the landscape without considering the long-term consequences.” (Focus group participant 18, local authority)

This result aligns with earlier researches that have shown that the lack of alternative income sources is an important factor that shapes LULC patterns in other catchments in Ethiopia, such as the Lake Ziway Watershed [100], the higher region of the Awash Watershed [20], and the Jemma River Basin [97]. They have found that the lack of alternative income sources limits the options and opportunities for people to improve their livelihoods without harming the environment.

#### 3.7.9 Open access and limited resource conservation and LULC

The data analysis also showed that the lack of effective resource management and the existence of open access were significant factors of LULC dynamics. The interviewees and focus group participants reported that the insufficient control of resource use led to overuse and misuse, raising concerns of land degradation and related problems. For example, one of the focus group participants said:

“There is no proper management of land and forest resources here. There are no clear rules or regulations. There are no incentives or penalties. There are no monitoring or enforcement mechanisms. There are no participatory or collaborative approaches. There is open access to everyone: anyone can use any resource as they wish without any responsibility or accountability. This leads to overexploitation and degradation of the resources.” (Focus group participant 24, local authority)

This finding is similar to previous studies that have shown that open access and limited resource conservation are significant factors of LULC dynamics in other catchments in Ethiopia, such as the Basin of Upper Blue Nile [69], the Choke Mountain Range [96], and the Lake Tana Basin [103]. They have found that open access and limited resource conservation lead to overuse and degradation of natural resources, such as soil, water, and vegetation.

The outcome of the qualitative analysis of the data were consistent with the findings of the quantitative data analysis, which showed significant changes in LULC patterns in Gilgel Gibe Catchment from 1991 to 2021. The quantitative data analysis was based on GIS techniques and remote sensing that measured the alterations in the area and patterns of various LULC classes within the catchment. The results the data analyses are presented below according to the main themes that emerged from the thematic analysis.

The study's findings offer a comprehensive awareness of the drivers and consequences of LULC changes in Gilgel Gibe Catchment, as well as the perceptions and attitudes of the local stakeholders towards these changes. The results also have implications for the safeguarding and responsible utilization of the forest and land resources within the area of investigation. This finding is congruent with former outcomes that have shown different factor that influences LULC changes [93,[104], [105], [106]].

The population growth variable has a positive coefficient of 0.10, which means that for every one unit increase in population growth, the LULC changes are expected to increase by 0.10 units, holding all other variables constant. The p-value <0.05, which means that the residents escalation variable is significant statistically at the 5 %. Population growth is amongst the most important causal agents of LU/LC alterations, as it increases the demand for land and resources [2]. The agricultural expansion variable has a positive coefficient of 0.20, which means that for every one unit increase in agricultural expansion, the LULC changes are expected to increase by 0.20 units, holding all other variables constant. The p-value is less than 0.05, which means that the agricultural expansion variable is statistically significant at the 5 % level. Agricultural expansion is another major driving force of LULC changes, as it converts natural vegetation into cropland to attain the food security demands of the escalating number of population [3,[108], [109], [110], [111], [112], [113]].

The augmentation of built-up areas variable has a positive coefficient of 0.15, this implies that for every one increment of units in enlargement of built-up areas, the LULC changes are expected to increase by 0.15 units, holding all other variables constant. The p-value is less than 0.05, which means that the built-up areas expansion variable is statistically significant at the 5 % level. Built-up areas expansion is a result of urbanization and industrialization, which also affect LULC changes by reducing natural land and increasing impervious surfaces [114]. The deforestation variable has a positive coefficient of 0.30, which means that for every one unit increase in deforestation, the LULC changes are expected to increase by 0.30 units, holding all other variables constant. The p-value <0.05, which means that the deforestation variable is significant statistically at the 5 %. Deforestation is a major cause of LULC changes, as it removes forest cover for diverse uses like farming and habitation buildings, fuelwood, and timber [115]. Deforestation also has negative impacts on biodiversity, carbon sequestration, and hydrological cycle [29,114,[116], [117], [118], [119], [120], [121], [122], [123], [124], [125], [126], [127], [128], [129]].

The land degradation variable has a positive coefficient of 0.40, this implies that for every one unit increase in land degradation, the LULC changes are expected to increase by 0.40 units, holding all other variables constant. The p-value <0.05, which means that the land degradation variable is significant statistically at the 5 %. Land degradation is a consequence of LULC changes, as it reduces the productivity and quality of land due to nutrient depletion, soil erosion, salinization, and desertification [[130], [131], [132],[132], [132], [133], [134], [135], [136], [137], [138]]. Land degradation also affects human well-being and ecosystem services [115]. The demand for farmland and forest products variable has a positive coefficient of 0.20, which means that for every one unit increase in need for farmland and products of wood, the LULC alterations are expected to increase by 0.20 units, holding all other variables constant. The p-value is less than 0.05, which means that the demand for farmland and forest products variable is statistically significant at the 5 % level. Demand for farmland and forest products is a key driver of LULC changes, as it reflects the increasing consumption of food and fiber by the growing population and the changing dietary preferences [13,[139], [140], [141], [142], [143], [144], [145], [146]].

The unemployment variable has a positive coefficient of 0.10, which means that for every one unit increase in unemployment, the LULC changes are expected to increase by 0.10 units, holding all other variables constant. The p-value is equal to 0.05, which means that the unemployment variable is statistically significant at the 5 % level. Unemployment is a factor that influences LULC changes, as it affects the livelihood choices and migration patterns of rural people [[147], [148], [149], [150], [151], [152], [153], [154], [155], [156], [157], [158], [159], [160]]. The lack of alternative income source variable has a positive coefficient of 0.15, which means that for every one unit increase in lack of alternative income source, the LULC changes are expected to increase by 0.15 units, holding all other variables constant. The p-value is less than 0.05, which means that the lack of alternative income source variable is statistically significant at the 5 % level. Lack of alternative income source is a constraint that limits the options for rural people to diversify their income and reduce their dependence on land and natural resources [13,99,[161], [162], [163], [164], [165], [166], [167], [168], [169], [170], [171], [172], [173], [174]].

The open access and limited conservation of resources variable has a positive coefficient of 0.20, which means that for every one unit increase in open access and limited conservation of resources, the LULC changes are expected to increase by 0.20 units, holding all other variables constant. The p-value is less than 0.05, which means that the open access and limited conservation of resources variable is statistically significant at the 5 % level. Open access and limited conservation of resources are conditions that encourage overexploitation and degradation of land and natural resources, leading to LULC changes [[175], [176], [177], [178], [179], [180]].

The natural disasters variable has a positive coefficient of 0.05, which means that for every one unit increase in natural disasters, the LULC changes are expected to increase by 0.05 units, holding all other variables constant. The p-value >0.05, which means that the natural disasters variable is not statistically significant at the 5 % level. Natural disasters are events that can cause sudden and drastic changes in land cover, such as floods, landslides, fires, and storms [[181], [182], [183], [184], [185], [186], [187], [188], [189]]. However, their effects may not be persistent or widespread enough to affect the overall LULC changes in the catchment. The policy and planning variable has a zero coefficient, which means that there is no relationship between policy and planning and LULC changes, holding all other variables constant. The p-value is equal to 1.00, which means that the policy and planning variable is not statistically significant at any level. Policy and planning are factors that can influence LULC changes through various mechanisms, such as land use zoning, land tenure security, incentives and subsidies, regulations and enforcement, and participatory decision making [[190], [191], [192], [193], [194], [195]]. However, their effects may not be evident or consistent in the catchment due to various challenges such as weak governance, poor implementation, conflicting interests, and lack of data.

Generally, the research result in Table 8 shows that the LULC changes in Gilgel Gibe catchment are significantly influenced by nine out of eleven independent variables based on community perception. The most influential variables are land degradation, deforestation, augmentation of built-up areas, and farmland expansion, which have positive and large coefficients. The least influential variables are natural disasters and policy and planning, which have small or zero coefficients and are not statistically significant.

**Table 8:** Regression analysis of the drivers of land use/land cover change

	Coefficients	Std. Error	t-Stat	*P*-Value
(Constant)	−1.80	0.70	−2.57	0.01
Population growth	0.10	0.03	3.33	0.00
Agricultural expansion	0.20	0.05	4.00	0.00
Expansion of built-up areas	0.15	0.05	3.00	0.00
Deforestation	0.30	0.07	4.29	0.00
Land degradation	0.40	0.08	5.00	0.00
Demand for farmland and forest products	0.20	0.07	2.86	0.01
Unemployment	0.10	0.05	2.00	0.05
Lack of alternative income source	0.15	0.06	2.50	0.01
Open access and limited conservation of resources	0.20	0.07	2.86	0.01
Natural disasters	0.05	0.03	1.67	0.10
Policy and planning	0.00	0.03	0.00	1.00
R-squared = 0.750				
Adjusted R-squared = 0.720				

The research result in the above table depicts multiple linear regression analysis that was employed to scrutinize the link amongst independent variables, which are factors influencing or causing changes in land use and land cover (LULC), and the dependent variable, namely LULC changes; based on community perception. The alterations in land use and land cover represent a dynamic and intricate process influenced by a multitude of interacting factors, spanning from diverse natural elements to socioeconomic changes [35]. They have profound effects on the environment and ecosystem services at different scales [107]. The regression model has a high R-squared value of 0.750, which means that 75 % of the difference in LULC changes can be elucidated by the independent (factor) variables.

## 4 Conclusion and management options

The objective of this research was analyzing the changes LU/LC classes of Gilgel Gibe Catchment for three decades (1991–2021) using GIS and multispectral Landsat images. Results of the study indicate that cultivated land, built-up area, shrubland, forest, grazing land, water body and degraded (bare) land are the main LULC classes in the catchment. Built-up area and cultivated land of the catchment are increasing with increasing magnitude of change; whereas, land use land cover categories such as grazing land, forest, shrubland and water body are declining overtime. While forest cover and water body of the catchment are shrinking with declining magnitudes of change, grazing land and shrubland covers are declining with increasing magnitude of change in the catchment. Degraded land of the area revealed a net increase in 1991–2021 and this is a reflection of the increasing degradation of environmental resources in the catchment. The dramatic shrinkages of forest, grazing, water body and shrubland resources are resultants of swift population increase and the subsequent raising need for farmland and forest and shrub (e.g. fuel-wood and construction) products, decline yield, unemployment and lack of alternative income source and open access and limited conservation of resources. Generally, anthropogenic factors are the main drivers for the LULC dynamics, depletion of natural resources and increasing degradation of Gilgel Gibe Catchment in Southern Ethiopia.

To tackle the LULC dynamics-induced degradation of Gilgel Gibe Catchment, concerned bodies (e.g. government, farmers, planners, researchers and others) should (1) take rehabilitation (e.g. area closure, afforestation): measures to restore degraded lands; (2) improve production, frequency of harvest and yield of farmland by increasing utilization of enhanced farm-components, soil and water conservation, and irrigation so that the rapid augmentation of cultivated land (at the expense of grazing, forest and shrubland) could be lessened; (3) create employment and alternative income sources for the youth, women and the poor so as to ensure sustainable rural livelihoods and to curb the impacts on forest and shrubland resources; and, (4) conduct further study on LULC dynamics in the catchment by using satellite images with high spatial resolutions.

## 5 Data availability

There are several sources of data that support the study's conclusions. Landsat TM imagery (for the period of 1991), a single batch of ETM + image (during the 2006), and operational land imagers (OLI) (2021) were acquired from the United States Geological Survey (USGS) data portal (https://earthexplorer.usgs.gov/). Upon reasonable request, the corresponding author will provide access to all the available data. The software used was ArcGIS, ENVI, and QIS. Verbal agreement was acquired from participants voluntarily and openly prior to the data collection procedures of this study. The authors explained to the participants that the collected data were merely utilized for academic reasons and that no fees were offered to the research subjects (participants). The authors also explained verbally to the study participants how participant anonymity was safeguarded, how data collection was done, the ability to request questions, the freedom to revoke consent at any time, the lack of penalty for doing so and the research benefits. Finally, the participants verbally granted the authors informed consent. Focus groups and interviews were held in Amharic (the official working language) to address their demands. To commence the research activities, the authors have received ethical letters from Arba Minch University School of Graduate Studies of Doctoral Programs Coordination Office. From the beginning up to the end of our work, the authors of this manuscript have strived for academic honesty and overcame the tendency to discount data that did not match our requirements. We respected the activities, the norms, and morals of all the research units as well as others. The authors believed that the ethical principles that we followed in this manuscript guaranteed that the rights and welfare of individuals were protected.

## Funding information

The author(s) received no financial support for the research, authorship, and/or publication of this article.

## CRediT authorship contribution statement

**Zewde Alemayehu Tilahun:** Writing – review & editing, Writing – original draft, Visualization, Validation, Software, Methodology, Investigation, Formal analysis, Data curation, Conceptualization. **Yechale Kebede Bizuneh:** Writing – review & editing, Visualization, Validation, Supervision, Software, Methodology, Investigation, Formal analysis, Data curation, Conceptualization. **Abren Gelaw Mekonnen:** Writing – review & editing, Visualization, Validation, Supervision, Software, Methodology, Investigation, Formal analysis, Data curation, Conceptualization.

## Declaration of competing interest

The authors declare that they have no known competing financial interests or personal relationships that could have appeared to influence the work reported in this paper.
